# Impact of aerobic exercise on cognitive function in patients with schizophrenia during daily nursing: A protocol for systematic review and meta-analysis

**DOI:** 10.1097/MD.0000000000023876

**Published:** 2021-01-08

**Authors:** Chunmian Chen, Yafang Yang, Xinwu Ye, Yulian Jin, Ziyao Cai, Jie Zheng

**Affiliations:** a Deanery; b Department of Nursing; c Ward 901; d Department of Sleep Medicine; e Department of Outpatient, Wenzhou Seventh People's Hospital, Wenzhou, P.R. China.

**Keywords:** aerobic exercise, meta-analysis, schizophrenia

## Abstract

**Objectives:**

To assess the effect of aerobic exercise (AE) on cognition function in people with schizophrenia (SZ) during daily nursing.

**Methods:**

The literature search will be conducted via PubMed, Embase, Cochrane Library, and Web of Science. Weighted mean difference (WMD) or standardized mean difference (SMD) and 95% confidence intervals (CIs) will be adopted to calculate the association between AE and cognitive function in patients with SZ. Publication bias will be performed by Begg test. When there is publication bias, “cut-and-fill method” will be adopted to adjust publication bias. Sensitivity analysis will be used to test the stability of the result. When the heterogeneity is large (*I*^2^ ≥ 50%), meta regression will be used to explore the source of inter-study heterogeneity. When the heterogeneity is large (*I*^2^ ≥ 50%) and the results are statistically significant (*P* < .05), age, sex, duration of disease, duration of intervention, amount of exercise per week, improvement of cardiopulmonary health, and other factors will be sub-analyzed.

**Conclusion:**

This meta-analysis will evaluate the impact of aerobic exercise on cognitive function in patients with SZ during daily nursing on the basis of existing evidence.

**OSF registration number:**

10.17605/OSF.IO/C8ABX

## 1 Introduction

Schizophrenia (SZ) is a severe mental illness that is characterized by cognitive impairment in areas of attention, working memory, and executive functioning, with a prevalence in the range of 0.7% to 1% worldwide.^[1–3]^ The recurrence rate of SZ within 2 or 3 years is about 40% and 70%, which leads to repeated or long-term hospitalization.^[4]^ Cognitive dysfunction is a main feature of SZ that primarily affects verbal learning and memory, attention, processing speed, and executive function, which not only brings severe psychological trauma and health threat to the patients and reduces their quality of life, but also brings great psychological and economic burden to their families.^[4–6]^ Thereby, it is necessary to improve cognitive impairment in patients with SZ, especially during daily nursing.

To ameliorate cognitive impairment, considerable efforts have been made to develop novel pharmacological, cognitive remediation approaches, and diversified rehabilitation treatments.^[7]^ At present, drug therapy is a main clinical practice for SZ patients.^[4,8,9]^ Nevertheless, adverse reactions of anti-psychotic drugs may contribute to an elevated risk of weight gain and the development of metabolic disturbances in SZ.^[3,10,11]^ Aerobic exercise (AE) is one of diversified rehabilitation strategies that has increasingly gained attention. Researches have confirmed the effects of exercise training on cognitive and brain plasticity in patients with neurological conditions like stroke, traumatic brain injury, and mild cognitive impairment.^[9,12–14]^ Multiple studies have shown that physical activity, and particularly structured exercise may significantly improve cognitive function in patients with SZ, particularly within domains of social cognition, working memory, processing speed, and attention.^[7,10,15,16]^

However, earlier studies of AE in SZ have not been able to determine the effects on cognition due to insufficient data.^[16]^ Given the results are inconsistent, herein we plan to present a meta-analysis to assess the effect of AE on cognition function in people with SZ during daily nursing.

## 2 Methods

The data were obtained from openly available databases, there were thus no need to get the approval from Institutional Review Board of our hospital.

### 2.1 Protocol registration

Prospective registration of this study has been approved by the Open Science Framework (OSF) registries (https://osf.io/registries), and the registration number is 10.17605/OSF.IO/C8ABX. This protocol for systematic review and meta-analysis was performed according to the Preferred Reporting Items for Systematic Review and Meta-Analysis Protocols (PRISMA-P) statement.

### 2.2 Search strategy

**Figure 1:**
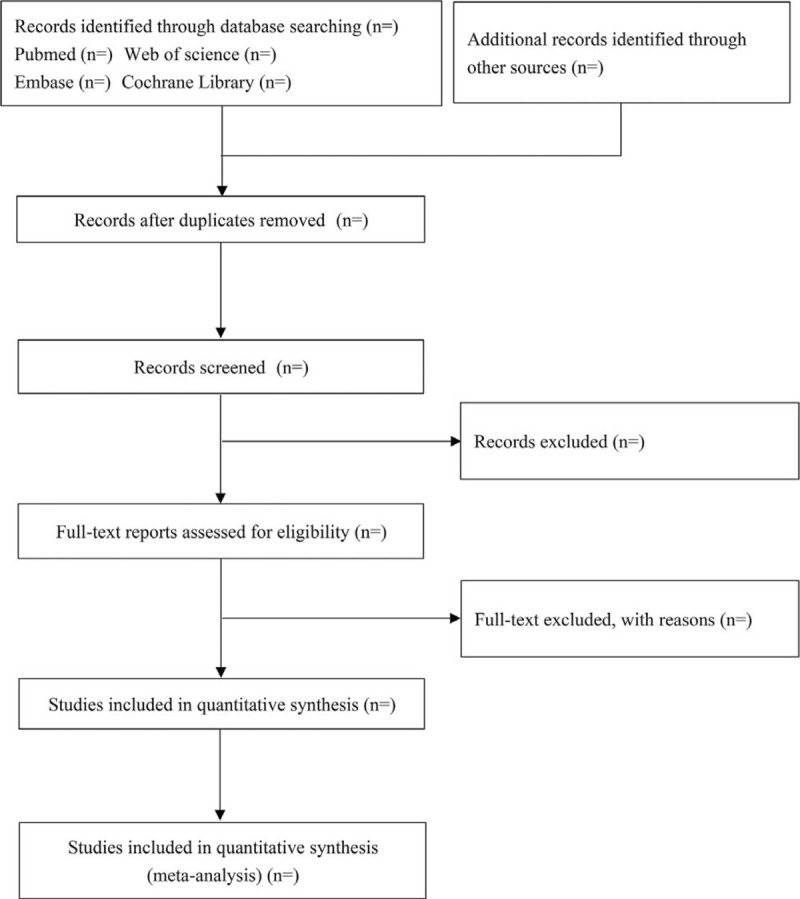
The diagram showing flow of study.

The literature search will be conducted via PubMed, Embase, Cochrane Library, and Web of Science. Search terms will be “Exercise” OR “Exercises” OR “Physical Activity” OR “Activities” OR “Physical” OR “Activity, Physical” OR “Physical Activities” OR “Exercise, Physical” OR “Exercises, Physical” OR “Physical Exercise” OR “Physical Exercises” OR “Acute Exercise” OR “Acute Exercises” OR “Exercise, Acute” OR “Exercises, Acute” OR “Exercise, Isometric” OR “Exercises, Isometric” OR “Isometric Exercises” OR “Isometric Exercise” OR “Exercise, Aerobic” OR “Aerobic Exercise” OR “Aerobic Exercises” OR “Exercises, Aerobic” OR “Exercise Training” OR “Exercise Trainings” OR “Training, Exercise” OR “Trainings, Exercise” AND “Schizophrenia” OR “Schizophrenias” OR “Schizophrenic Disorders” OR “Disorder, Schizophrenic” OR “Disorders, Schizophrenic” OR “Schizophrenic Disorder” OR “Dementia Praecox” AND “Cognition” OR “Cognitions” OR “Cognitive Function” OR “Cognitive Functions” OR “Function, Cognitive” OR “Functions, Cognitive”. The search strategy is displayed in Table 1, and the diagram showing flow of study is shown in Fig. 1.

**Table 1:** PubMed search strategy.

No.	Search items
#1	(((((((((((((((((((((((((Exercise[Mesh Terms]) OR (Exercises[Title/Abstract])) OR (Physical Activity[Title/Abstract])) OR (Activities, Physical[Title/Abstract])) OR (Activity, Physical[Title/Abstract])) OR (Physical Activities[Title/Abstract])) OR (Exercise, Physical[Title/Abstract])) OR (Exercises, Physical[Title/Abstract])) OR (Physical Exercise[Title/Abstract])) OR (Physical Exercises[Title/Abstract])) OR (Acute Exercise[Title/Abstract])) OR (Acute Exercises[Title/Abstract])) OR (Exercise, Acute[Title/Abstract])) OR (Exercises, Acute[Title/Abstract])) OR (Exercise, Isometric[Title/Abstract])) OR (Exercises, Isometric[Title/Abstract])) OR (Isometric Exercises[Title/Abstract])) OR (Isometric Exercise[Title/Abstract])) OR (Exercise, Aerobic[Title/Abstract])) OR (Aerobic Exercise[Title/Abstract])) OR (Aerobic Exercises[Title/Abstract])) OR (Exercises, Aerobic[Title/Abstract])) OR (Exercise Training[Title/Abstract])) OR (Exercise Trainings[Title/Abstract])) OR (Training, Exercise[Title/Abstract])) OR (Trainings, Exercise[Title/Abstract])
#2	((((((Schizophrenia[Mesh Terms]) OR (Schizophrenias[Title/Abstract])) OR (Schizophrenic Disorders[Title/Abstract])) OR (Disorder, Schizophrenic[Title/Abstract])) OR (Disorders, Schizophrenic[Title/Abstract])) OR (Schizophrenic Disorder[Title/Abstract])) OR (Dementia Praecox[Title/Abstract])
#3	(((((Cognition[Mesh Terms]) OR (Cognitions[Title/Abstract])) OR (Cognitive Function[Title/Abstract])) OR (Cognitive Functions[Title/Abstract])) OR (Function, Cognitive[Title/Abstract])) OR (Functions, Cognitive[Title/Abstract])
#4	#1 AND #2 AND #3

### 2.3 Study selection

Inclusion criteria: patients with SZ; experimental group: patients who received AE (such as walking, running, aerobics, and cycling), and the control group: patients treated with occupational therapy (OT), treatment as usual (TAU), table football, cognitive remediation (CR), or waitlist; randomized controlled trials (RCTs) and non-RCTs; English articles.

Excluded criteria: patients with depression, mania, and attention deficit hyperactivity disorder (ADHD); animal experiments; Yoga and Tai chi; researches that are not relevant to the subject; study in which data are incomplete or cannot be extracted; case reports, editing materials, meeting abstracts, reviews, and meta-analyses.

### 2.4 Methodological quality appraisal and data extraction

The improved Jadad scale will be used to evaluate the quality of the RCTs with a total score of 7, 1 to 3 being of low quality and 4 to 7 being of high quality. For the non-RCTs, MINORS scale will be adopted. The total score is 24 scores, 1 to 12 scores as low quality, and 13 to 24 scores as high quality.

The data will be extracted independently by 2 authors (YY and XY), which containing the first author name, year of publication, the country where the study was conducted, the study design, the data source of the selected patients or study participants, the intervention and control methods, the number of patients in the intervention/control group, age, sex ratio, follow-up time, etc. If information is lost, an attempt will be made to contact the original study author for relevant information. If not, the missing information will be calculated from the relevant data in the study if possible. The data extraction tables of the 2 authors (YY and XY) will be cross-checked to verify the accuracy and consistency of the extracted data. Any differences in the above process will be resolved through discussion between 2 authors (YY and XY), and any further differences will be arbitrated by a third author (CC).

### 2.5 Assessment indicators

The impact of AE on cognitive function in patients with SZ during daily nursing will be assessed on the basis of 3 main indicators.

#### 2.5.1 Global cognition

This is defined as average change in all clinically validated measures of cognitive functioning following an exercise intervention.^[16]^ In the case of reporting changes in multiple cognitive tasks/domains, the composite change score will be calculated based on the average change in each task/cognitive domain.

#### 2.5.2 Individual cognitive domains

Evaluate the role of AE in the field of individual cognition according to the categories established in the Measurement and Treatment Research to Improve Cognition in SZ (MATRICS) developed by the National Institute of Mental Health (NIMH). The 7 specific categories of cognition specified are speed of processing, attention/vigilance, working memory, verbal learning and memory, visual learning and memory, reasoning and problem solving, and social cognition.

#### 2.5.3 Potential influencing factors

Data on factors which may influence the effect size of exercise interventions will also be extracted from each study, including sample characteristics (age, sex distribution, years of illness duration), exercise intervention characteristics (minutes of exercise per week, maximal oxygen uptake, professional background of instructor), and study design (control condition will be used and trial quality).

### 2.6 Statistical analysis

Stata15.1 software (Stata Corporation, College Station, TX) will be used for statistical analysis. If the same assessment tool is used for measurement data, weighted mean difference (WMD) will be adopted as the effect indicator, and no-standard method will be adopted to combine the data. If different evaluation tools are used for measurement data, standardized mean difference (SMD) will be used as the effect indicator, and the Hedges method will be used to merge the data. Each effect size will be expressed by 95% confidence intervals (CIs), and *P* < .05 will be considered statistically significant. Publication bias will be performed by Begg test. When there is publication bias, “cut-and-fill method” will be adopted to adjust publication bias. All assessment indicators will be subjected to sensitivity analysis to test the stability of the result. When the heterogeneity is large (*I*^2^ ≥ 50%), meta regression will be used to explore the source of inter-study heterogeneity. When the heterogeneity is large (*I*^2^ ≥ 50%) and the results are statistically significant (*P* < .05), age, sex, duration of disease, duration of intervention, amount of exercise per week, improvement of cardiopulmonary health, and other factors will be sub analyzed.

## 3 Discussion

SZ patients tend to be more sedentary compared with general population,^[17,18]^ but increase the level of physical activity of these patients seem essential, and also an easy method for preventing, minimizing the many health problems associated with sedentary lifestyle and reduced mortality.^[19]^ AE can improve the level of central nervous system function, promote the body to adapt to the external environment, so as to effectively relieve negative emotions and avoid neurasthenia; At the same time, it can enhance the patients’ sense of social participation, generate strong pleasure, confidence, and sense of achievement, so as to improve their confidence in rehabilitation. In addition, appropriate AE can improve human mental agility and regulate brain attention, so as to significantly improve patients’ cognitive function.^[8]^ AE nursing is a kind of comprehensive nursing intervention measures.^[20]^ Daily family nursing of AE plays an important role in the recovery of the diseases, such as chronic obstructive pulmonary disease,^[20]^ coronary heart disease,^[21]^ which not only reduces the recurrence of the diseases, but also effectively accelerate the rehabilitation process.^[22]^ Thus, it is important to assess the impact of AE on cognitive function in patients with SZ in daily nursing.

Studies have shown the enhancement in composite cognitive test scores,^[23]^ hippocampal volume, short-term memory,^[24]^ working memory, and speed of processing^[25]^ in patients with SZ after AE training. A quasi-experimental study^[26]^ explored the effects of AE on patients with a 10-year history of taking psychotropic drugs. The study found that hallucinations and delusions symptoms were relieved after 10 weeks of AE treatment for 40 minutes per day, 3 times a week. At the same time, in the cognitive analogy stage after training, there was no significant decline in the amount of thoughts, language fluency, and productivity.^[27]^ Nevertheless, Biddle^[28]^ pointed out physical activity does not help people with SZ by reducing their cognitive impairment, but simply improves their quality of life by arranging their environmental conditions to reduce anxiety, depression, and increase self-confidence.^[26]^

Study found that cognitive improvement is related to the length of exercise.^[29]^ Firth et al^[16]^ found that higher weekly duration of AE may be associated with greater improvement in cognition among people with SZ, particularly from interventions using higher dosages of exercise, which were supervised by physical activity professionals. Similarly, the study of Chen et al^[10]^ also indicated patients with SZ who spent more time in light physical activity showed better performance on attention/concentration and speed of processing tasks than those who were less active. Kimhy et al^[30]^ and Leutwyler et al^[29]^ revealed the similar findings that high physical fitness or more physical activity are associated with better cognitive performance, including executive functioning, verbal working memory, and speed of processing among patients with SZ. However, Kimhy et al^[31]^ previously examined the relative influence of exercise duration, frequency, and intensity on cognitive improvements following a 12-week exercise program in SZ and found that intensity was the best predictor variable.

Cognitive impairment is the core symptom of SZ and is one of the effective indicators to predict the treatment effect of patients. It is necessary to improve patients’ cognitive function through effective intervention. AE therapy may reduce the adverse reactions caused by drug therapy, which may better improve patient's cognitive function. While some results also suggested AE can’t improve the cognitive function in SZ patients. Since the result is controversial, we will perform this study to investigate the impact of AE on cognitive function in patients with SZ during daily nursing. We will comprehensively search, screen, assess, and extract valuable data from several databases as previously mentioned, and report this review results according to the PRISMA guidelines. To our knowledge, this is the first meta-analysis to demonstrate the impact of AE on SZ patients during daily nursing.

## Author contributions

**Conceptualization:** Chunmian Chen, Jie Zheng.

**Data curation:** Chunmian Chen, Yafang Yang, Xinwu Ye, Yulian Jin, Ziyao Cai, Jie Zheng.

**Supervision:** Jie Zheng.

**Visualization:** Yulian Jin.

**Writing – original draft:** Chunmian Chen.

**Writing – review & editing:** Jie Zheng.
